# Using V̇o_2max_ as a marker of training status in athletes—can we do better?

**DOI:** 10.1152/japplphysiol.00723.2021

**Published:** 2022-02-17

**Authors:** Tim Podlogar, Peter Leo, James Spragg

**Affiliations:** ^1^School of Sport, Exercise and Rehabilitation Sciences, University of Birmingham, Birmingham, United Kingdom; ^2^Faculty of Health Sciences, University of Primorska, Izola, Slovenia; ^3^Human Performance Centre, Ljubljana, Slovenia; ^4^Division of Performance Physiology & Prevention, Department of Sports Science, University of Innsbruck, Innsbruck, Austria; ^5^Health through Physical Activity, Lifestyle and Sports (HPALS) Research Centre, Faculty of Health Sciences, University of Cape Town, Cape Town, South Africa

**Keywords:** elite athletes, endurance, maximal oxygen uptake, maximal power output, performance prediction

## INTRODUCTION

**Figure 1.**
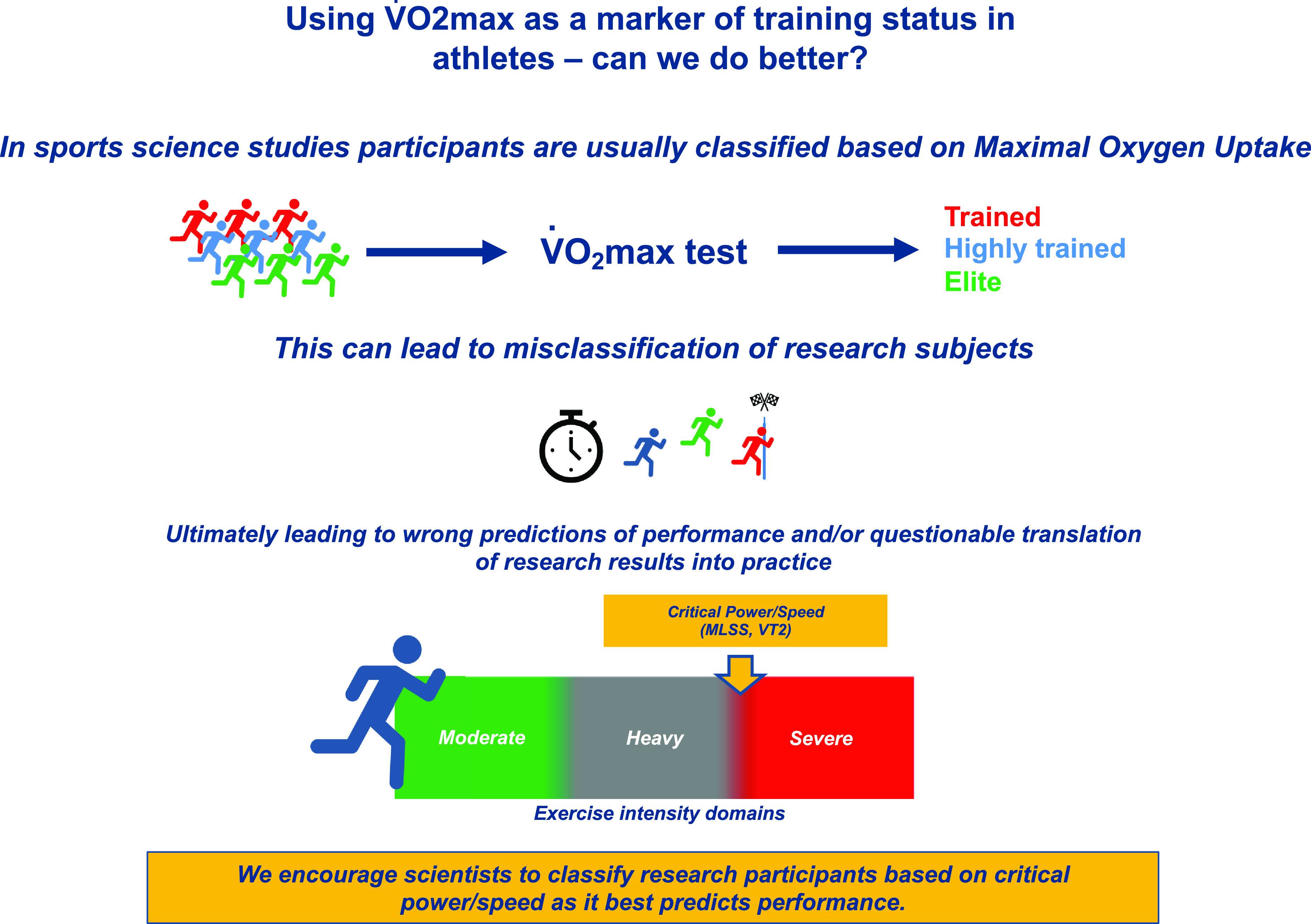
Classification of research study participants based on maximal oxygen uptake can lead to questionable translation of research results into practice given that maximal oxygen uptake is not a good predictor of elite endurance performance.

One of the fundamental premises of research is that findings from a sample of a population can be extrapolated to the population at large. Therefore, correct classification of the sample population is of paramount importance, especially as it has been shown that there is a nonuniform response to the same intervention in athletes of differing training statuses (1, 2); for example, nitrate supplementation was thought to be beneficial based on research in nonelite populations; however, these findings have not been replicated in elite populations (3). If a strategy described in a research study is to be applied in real-world athletes, the expected outcomes should be similar. However, as demonstrated, this may not be the case if the research study participants are not correctly classified. In sports science studies, participants are most commonly classified based on their maximal oxygen uptake (V̇o_2max_) and the maximal power at the end of a laboratory-based graded exercise test (Wmax). The aim of this viewpoint is to present the argument that the training status of participants could be better defined. To this end, we suggest that the power/speed at the boundary of the heavy/severe exercise intensity domain should be reported as the main descriptor of training status in studies where the readership may be interested in the performance implications of a given intervention (Fig. 1).

### Limitations of Current Classification Practices

Measuring and reporting relative and absolute V̇o_2max_ values has a long history in the field of exercise sciences because it not only offers a prognosis of health outcomes and mortality but also is more pertinent to the present article; it is believed that normalization of V̇o_2max_ to body mass is a good predictor of performance, and thus a broad descriptor of the training status. As a result, V̇o_2max_ is used to describe study participants. There is no doubt that V̇o_2max_ is a solid predictor of endurance performance in a heterogeneous group of participants (4). However, using V̇o_2max_ as the primary determinant of participant classification has led to some common issues within the literature (5).

First, there can be a mismatch between the actual performance level of athletes and their classification based on their V̇o_2max_ values. For instance, participants have been classified as *elite* despite not even competing at the lowest international level (6, 7). Second, V̇o_2max_ alone does not predict differences in performance in a relatively homogenous group (13). This is elegantly demonstrated by the nonsignificant differences in V̇o_2max_ in a group of U23 professional cyclists (9) despite differences in their level of performance. Third, reported V̇o_2max_ in Olympians, professional athletes, and world record holders would have them classified into inferior categories based purely on V̇o_2max_ (8, 10, 11). Finally, there can be large discrepancies in V̇o_2max_ between athletes with similar performance capabilities, for example, V̇o_2max_ in world-class marathon runners can differ by up to 22 mL·kg^−1^·min^−1^ (10).

Differences in actual performance between athletes may therefore be related to additional factors (12). First, exercise economy/efficiency; this parameter describes how well oxygen is converted into locomotion at submaximal intensities and has been shown to be significantly different between groups with nonsignificant differences in V̇o_2max_ (13). It has also been shown that V̇o_2max_ is inversely associated with running economy (10), indicating that V̇o_2max_ per se cannot independently predict performance. Second, interindividual differences in the maximal sustainable fractional utilization of V̇o_2max_ (%V̇o_2max_) (14); even though alone this variable does not always account for differences in performance (15).

Combined these findings show that V̇o_2max_ can only be used as a descriptor and a predictor of performance when other factors are also reported (16, 41). This is well demonstrated as athletes have been shown to improve their performance irrespective of an increase in V̇o_2max_ (17, 18).

Recently, a new framework for classification of study participants has been published (2). This work highlights similar drawbacks in current practice to those presented here. It proposes a new classification system based primarily on training norms and competition results. Although we are supportive of the ideas presented in this article, the advantage and disadvantage of this approach is that competitive results are an aggregate of various factors (e.g., psychology, tactical skills) and not necessarily just physiology. We believe that in addition to describing competitive status, classifying participants based on their performance physiology provides an additional layer of information that is useful from both an academic and applied perspective.

A pertinent solution could be the application of an external measure that predicts performance and is a product of the aforementioned underlying physiological parameters (18). The suitability of external measures to discriminate between athletes can be demonstrated using two cycling case studies, one of a multiple grand tour winner (20) and one of an athlete with the highest ever recorded V̇O_2max_ (21). Comparison reveals that even having the highest V̇o_2max_ is no guarantee of success. It also highlights that describing participants according to an external measure, in this case, power at a blood lactate concentration of 4 mmol·L^−1^, is more revealing than V̇o_2max_. Namely, the multiple grand tour winner had a lower V̇o_2max_ but displayed higher power at a lactate concentration of 4 mmol·L^−1^.

### Power/Speed at the Boundary of the Heavy and Severe Exercise-Intensity Domains

An enticing option to classifying study participants (in endurance sports) would be to use the power/speed at the boundary of the heavy and severe exercise-intensity domains. This approach demonstrated a high practical utility in predicting endurance performance (22) and can differentiate between performance in athletes with similar V̇o_2max_ (23). The demarcating intensity between the two domains has been described as critical power (CP), critical speed (CS), maximal lactate steady state (MLSS), or the second ventilatory threshold (VT2) (24). Although all three represent physiological landmarks occurring at a similar exercise intensity, the current weight of evidence points toward the CP/CS model offering the most comprehensive explanation of performance over various exercise durations (25–29). It has also been suggested that the CP/CS best represents the threshold between steady and nonsteady exercise (24, 30, 31); however, the arguments surrounding this topic are outside the scope of this viewpoint (32, 33).

We, therefore, propose that CP/CS rather than V̇o_2max_ should be used as the primary descriptor of participants’ training status.

CP/CS was first described as the asymptote of the curvilinear relationship between power/speed and time to task failure (34). Subsequent developments in the understanding of the mechanistic basis of the CP/CS means it is currently understood to be the maximum power or speed at which there is no metabolite-induced progressive derangement of muscle cell homeostasis (35). By using the CP/CS concept, one can also calculate the fixed work capacity above the CP/CS (W’ or D’). W’/D’ represents a fixed work capacity above the CP/CS that can be utilized within the severe exercise-intensity domain (30). Using the CP/CS and W’/D’ together it is possible to predict performance in shorter events (28, 36).

Thus, CP/CS, accompanied by the W’/D’, arguably gives an insight into performance capacity across a wider range of durations and exercise modalities than either V̇o_2max_ or Wmax (28), or indeed any other measure of the heavy/severe exercise-intensity domain border (37). Indeed, the CP concept has been applied to predict performance across exercise durations from single repetition maximum (29) to marathon performance (27).

### Additional Benefits of Using Critical Power/Speed to Determine Participant Status

Although there are methodical issues associated with deriving CP/CS (38), the authors believe that if recognized guidelines are applied, valid CP/CS estimates can be easily obtained. CP/CS estimates can easily be derived in both formal laboratory and field-based testing (9, 39) without the use of specialized equipment. Due to the ease of determination, practitioners can easily derive CP/CS in their own athlete populations and compare these values with those in a given study to judge whether an intervention is warranted and allow a better prediction of the magnitude of potential performance improvements.

V̇o_2max_ and Wmax are also often used in studies to determine exercise intensity in subsequent interventions. However, this approach is flawed, as there are interindividual differences in the percentage of V̇o_2max_ and Wmax at which boundaries between different exercise intensity domains occur. Thus, different physiological responses between participants can be observed when anchoring exercise intensity to fractions of V̇o_2max_ or Wmax (40). If the CP/CS is determined as part of the classification process, these values can also be used to anchor exercise intensity in any subsequent intervention.

## CONCLUSIONS

Based on the arguments above, it is the authors’ opinion that researchers should be encouraged to describe study participants based on the physiological parameters capable of best predicting performance across a wide range of intensities and to move away from reporting solely V̇o_2max_. It is our belief that application of the CP/CS concept would provide the most appropriate way to do this.

## DISCLOSURES

No conflicts of interest, financial or otherwise, are declared by the authors.

## AUTHOR CONTRIBUTIONS

T.P., P.L., and J.S. drafted manuscript; T.P., P.L., and J.S. edited and revised manuscript; T.P., P.L., and J.S. approved final version of manuscript.

## References

[1] McConell GK, Wadley GD, Le Plastrier K, Linden KC. Skeletal muscle AMPK is not activated during 2 h of moderate intensity exercise at ∼65% V̇o_2peak_ in endurance trained men. J Physiol 598: 3859–3870, 2020. doi:10.1113/JP277619. 32588910 PMC7540472

[2] McKay AKA, Stellingwerff T, Smith ES, Martin DT, Mujika I, Goosey-Tolfrey VL, Sheppard J, Burke LM. Defining training and performance caliber: a participant classification framework. Int J Sports Physiol Perform 17: 317–331, 2022. doi:10.1123/ijspp.2021-0451. 34965513

[3] Jonvik KL, Nyakayiru J, Van Loon LJC, Verdijk LB. Can elite athletes benefit from dietary nitrate supplementation? J Appl Physiol (1985) 119: 759–761, 2015. doi:10.1152/japplphysiol.00232.2015. 25997946

[4] Magel JR, Faulkner JA. Maximum oxygen uptakes of college swimmers. J Appl Physiol 22: 929–933, 1967. doi:10.1152/jappl.1967.22.5.929. 6025749

[5] Coyle EF, Coggan AR, Hopper MK, Walters TJ. Determinants of endurance in well-trained cyclists. J Appl Physiol (1985) 64: 2622–2630, 1988. doi:10.1152/jappl.1988.64.6.2622. 3403447

[6] Gejl KD, Thams LB, Hansen M, Rokkedal-Lausch T, Plomgaard P, Nybo L, Larsen FJ, Cardinale DA, Jensen K, Holmberg HC, Vissing K, Ørtenblad N. No superior adaptations to carbohydrate periodization in elite endurance athletes. Med Sci Sports Exerc 49: 2486–2497, 2017. doi:10.1249/MSS.0000000000001377. 28723843

[7] Bardis CN, Kavouras SA, Adams JD, Geladas ND, Panagiotakos DB, Sidossis LS. Prescribed drinking leads to better cycling performance than ad libitum drinking. Med Sci Sports Exerc 49: 1244–1251, 2017. doi:10.1249/MSS.0000000000001202. 28079705

[8] Burke LM, Ross ML, Garvican-Lewis LA, Welvaert M, Heikura IA, Forbes SG, Mirtschin JG, Cato LE, Strobel N, Sharma AP, Hawley JA. Low carbohydrate, high fat diet impairs exercise economy and negates the performance benefit from intensified training in elite race walkers. J Physiol 595: 2785–2807, 2017. doi:10.1113/JP273230. 28012184 PMC5407976

[9] Leo P, Spragg J, Simon D, Lawley JS, Mujika I. Training characteristics and power profile of professional U23 cyclists throughout a competitive season. Sports (Basel) 8: 167, 2020. doi:10.3390/sports8120167. 33348618 PMC7766290

[10] Jones AM, Kirby BS, Clark IE, Rice HM, Fulkerson E, Wylie LJ, Wilkerson DP, Vanhatalo A, Wilkins BW. Physiological demands of running at 2-hour marathon race pace. J Appl Physiol (1985) 130: 369–379, 2021. doi:10.1152/japplphysiol.00647.2020. 33151776

[11] Leckey JJ, Ross ML, Quod M, Hawley JA, Burke LM. Ketone diester ingestion impairs time-trial performance in professional cyclists. Front Physiol 8: 806, 2017. doi:10.3389/fphys.2017.00806. 29109686 PMC5660098

[12] Coyle EF, Coggan AR, Hopper K, Walters TJ. Determinants of endurance in well-trained cyclists. J Appl Physiol (1985) 64: 2622–2630, 1988. doi:10.1152/jappl.1988.64.6.2622.[3403447] 3403447

[13] Sallet P, Mathieu R, Fenech G, Baverel G. Physiological differences of elite and professional road cyclists related to competition level and rider specialization. J Sports Med Phys Fitness 46: 361–365, 2006. 16998438

[14] Billat V, Bernard O, Pinoteau J, Petit B, Koralsztein JP. Time to exhaustion at V̇o_2max_ and lactate steady state velocity in sub elite long-distance runners. Arch Int Physiol Biochim Biophys 102: 215–219, 1994. doi:10.3109/13813459409007541. 8000045

[15] Støa EM, Støren Ø, Enoksen E, Ingjer F. Percent utilization of V̇O_2_ max at 5-km competition velocity does not determine time performance at 5 km among elite distance runners. J Strength Cond Res 24: 1340–1345, 2010. doi:10.1519/JSC.0b013e3181cc5f7b. 20386483

[16] Joyner MJ. Modeling: optimal marathon performance on the basis of physiological factors. J Appl Physiol (1985) 70: 683–687, 1991. doi:10.1152/jappl.1991.70.2.683. 2022559

[17] Jones AM. A five year physiological case study of an Olympic runner. Br J Sports Med 32: 39–43, 1998. doi:10.1136/bjsm.32.1.39. 9562162 PMC1756052

[18] McLaughlin JE, Howley ET, Bassett DR Jr, Thompson DL, Fitzhugh EC. Test of the classic model for predicting endurance running performance. Med Sci Sports Exerc 42: 991–997, 2010. doi:10.1249/MSS.0b013e3181c0669d. 19997010

[20] Bell PG, Furber MJW, VAN Someren K, Antón-Solanas A, Swart J. The physiological profile of a multiple Tour de France winning cyclist. Med Sci Sports Exerc 49: 115–123, 2017. doi:10.1249/MSS.0000000000001068. 27508883

[21] Rønnestad BR, Hansen J, Stensløkken L, Joyner MJ, Lundby C. Case Studies in Physiology: Temporal changes in determinants of aerobic performance in individual going from alpine skier to world junior champion time trial cyclist. J Appl Physiol (1985) 127: 306–311, 2019. doi:10.1152/japplphysiol.00798.2018. 31194601

[22] Nimmerichter A, Prinz B, Gumpenberger M, Heider S, Wirth K. Field-derived power-duration variables to predict cycling time-trial performance. Int J Sports Physiol Perform 15: 1095–1102, 2020. doi:10.1123/IJSPP.2019-0621. 32040941

[23] Loftin M, Warren B. Comparison of a simulated 16.1-km time trial, V̇o_2max_ and related factors in cyclists with different ventilatory thresholds. Int J Sports Med 15: 498–503, 1994. doi:10.1055/s-2007-1021094. 7890464

[24] Poole DC, Rossiter HB, Brooks GA, Gladden LB. The anaerobic threshold: 50+ years of controversy. J Physiol 599: 737–767, 2021. doi:10.1113/JP279963. 33112439

[25] Vanhatalo A, Jones AM, Burnley M. Application of critical power in sport. Int J Sports Physiol Perform 6: 128–136, 2011. doi:10.1123/ijspp.6.1.128. 21487156

[26] Black MI, Durant J, Jones AM, Vanhatalo A. Critical power derived from a 3-min all-out test predicts 16.1-km road time-trial performance. Eur J Sport Sci 14: 217–223, 2014. doi:10.1080/17461391.2013.810306. 23802599

[27] Florence SI, Weir JP. Relationship of critical velocity to marathon running performance. Eur J Appl Physiol Occup Physiol 75: 274–278, 1997. doi:10.1007/s004210050160. 9088849

[28] Kirby BS, Winn BJ, Wilkins BW, Jones AM. Interaction of exercise bioenergetics with pacing behavior predicts track distance running performance. J Appl Physiol (1985) 131: 1532–1542, 2021. doi:10.1152/japplphysiol.00223.2021. 34617823

[29] Morton RH, Redstone MD, Laing D. The critical power concept and bench press: modeling 1RM and repetitions to failure. Int J Exerc Scie 7: 152–160, 2014. https://digitalcommons.wku.edu/ijes/vol7/iss2/6

[30] Jones AM, Vanhatalo A, Burnley M, Morton RH, Poole DC. Critical power: implications for determination of V̇o_2max_ and exercise tolerance. Med Sci Sports Exerc 42: 1876–1890, 2010. doi:10.1249/MSS.0b013e3181d9cf7f. 20195180

[31] Poole DC, Burnley M, Vanhatalo A, Rossiter HB, Jones AM. Critical power: an important fatigue threshold in exercise physiology. Med Sci Sports Exerc 48: 2320–2334, 2016. doi:10.1249/MSS.0000000000000939. 27031742 PMC5070974

[32] Jones AM, Burnley M, Black MI, Poole DC, Vanhatalo A. The maximal metabolic steady state: redefining the “gold standard’’. Physiol Rep 7: e14098, 2019. doi:10.14814/phy2.14098. 31124324 PMC6533178

[33] Mattioni Maturana F, Keir DA, McLay KM, Murias JM. Can measures of critical power precisely estimate the maximal metabolic steady-state? Appl Physiol Nutr Metab 41: 1197–1203, 2016. doi:10.1139/apnm-2016-0248. 27819154

[34] Monod H, Scherrer J. The work capacity of a synergic muscular group. Ergonomics 8: 329–338, 1965. doi:10.1080/00140136508930810.

[35] Jones AM, Wilkerson DP, DiMenna F, Fulford J, Poole DC. Muscle metabolic responses to exercise above and below the “critical power” assessed using 31P-MRS. Am J Physiol Regul Integr Comp Physiol 294: R585–R593, 2008. doi:10.1152/ajpregu.00731.2007. 18056980

[36] Morgan PT, Black MI, Bailey SJ, Jones AM, Vanhatalo A. Road cycle TT performance: relationship to the power-duration model and association with FTP. J Sports Sci 37: 902–910, 2019. doi:10.1080/02640414.2018.1535772. 30387374

[37] Leo P, Spragg J, Podlogar T, Lawley JS, Mujika I. Power profiling and the power-duration relationship in cycling: a narrative review. Eur J Appl Physiol 122: 301–316, 2022. doi:10.1007/s00421-021-04833-y. 34708276 PMC8783871

[38] Mattioni Maturana F, Fontana FY, Pogliaghi S, Passfield L, Murias JM. Critical power: how different protocols and models affect its determination. J Sci Med Sport 21: 742–747, 2018. doi:10.1016/j.jsams.2017.11.015. 29203319

[39] Karsten B, Jobson SA, Hopker J, Stevens L, Beedie C. Validity and reliability of critical power field testing. Eur J Appl Physiol 115: 197–204, 2015. doi:10.1007/s00421-014-3001-z. 25260244

[40] Jamnick NA, Pettitt RW, Granata C, Pyne DB, Bishop DJ. An examination and critique of current methods to determine exercise intensity. Sports Mede 50: 1729–1756, 2020. doi:10.1007/s40279-020-01322-8. 32729096

[41] Legaz-Arrese A, Munguía-Izquierdo D, Nuviala Nuviala A, Serveto-Galindo O, Moliner Urdiales D, Reverter Masía J. Average V̇o_2max_ as a function of running performances on different distances. Sci Sports 22: 43–49, 2007. doi:10.1016/j.scispo.2006.01.008.

